# *Pv*RON2, a new *Plasmodium vivax *rhoptry neck antigen

**DOI:** 10.1186/1475-2875-10-60

**Published:** 2011-03-14

**Authors:** Gabriela Arévalo-Pinzón, Hernando Curtidor, Liliana C Patiño, Manuel A Patarroyo

**Affiliations:** 1 Fundación Instituto de Inmunología de Colombia FIDIC, Carrera 50 # 26-20, Bogotá, Colombia; 2 Universidad del Rosario, Carrera 24 # 63C-69, Bogotá, Colombia; 3 Pontificia Universidad Javeriana, Carrera 7 # 40-62, Bogotá, Colombia

## Abstract

**Background:**

Rhoptries are specialized organelles from parasites belonging to the phylum *Apicomplexa*; they secrete their protein content during invasion of host target cells and are sorted into discrete subcompartments within rhoptry neck or bulb. This distribution is associated with these proteins' role in tight junction (TJ) and parasitophorous vacuole (PV) formation, respectively.

**Methods:**

*Plasmodium falciparum *RON2 amino acid sequence was used as bait for screening the codifying gene for the homologous protein in the *Plasmodium vivax *genome. Gene synteny, as well as identity and similarity values, were determined for *ron2 *and its flanking genes among *P. falciparum*, *P. vivax *and other malarial parasite genomes available at PlasmoDB and Sanger Institute databases. *Pvron2 *gene transcription was determined by RT-PCR of cDNA obtained from the *P. vivax *VCG-1 strain. Protein expression and localization were assessed by Western blot and immunofluorescence using polyclonal anti-*Pv*RON2 antibodies. Co-localization was confirmed using antibodies directed towards specific microneme and rhoptry neck proteins.

**Results and discussion:**

The first *P. vivax *rhoptry neck protein (named here *Pv*RON2) has been identified in this study. *Pv*RON2 is a 2,204 residue-long protein encoded by a single 6,615 bp exon containing a hydrophobic signal sequence towards the amino-terminus, a transmembrane domain towards the carboxy-terminus and two coiled coil α-helical motifs; these are characteristic features of several previously described vaccine candidates against malaria. This protein also contains two tandem repeats within the interspecies variable sequence possibly involved in evading a host's immune system. *Pv*RON2 is expressed in late schizonts and localized in rhoptry necks similar to what has been reported for *Pf*RON2, which suggests its participation during target cell invasion.

**Conclusions:**

The identification and partial characterization of the first *P. vivax *rhoptry neck protein are described in the present study. This protein is homologous to *Pf*RON2 which has previously been shown to be associated with *Pf*AMA-1, suggesting a similar role for *Pv*RON2.

## Background

Of the five *Plasmodium *parasite species producing human malaria, *Plasmodium vivax *causes 100 to 300 million clinical cases per year [1,2], representing ~40% of the population suffering from this disease. Although *P. vivax *malaria has been considered to be less severe than that produced by *Plasmodium falciparum *in clinical terms, several factors have highlighted the need to search for new effective control measures to counteract *P. vivax *infections, i.e. its ability to cause chronic infections by inducing dormant forms present in the liver (hypnozoites), increased severe manifestations caused by this parasite species and the emergence of strains resistant to chemotherapeutic agents, such as chloroquine [3,4]. Due to the difficulty of carrying out a *P. vivax *continuous culture *in vitro*, this parasite has been relatively less studied compared to other *Plasmodium *species. To overcome this problem, a comparative approach has been undertaken aimed at identifying and characterizing in *P. vivax *parasite molecules involved in target cell invasion previously described for other *Plasmodium *species (mainly *P. falciparum*), and in recent transcriptome studies of the *P. vivax *intraerythrocytic development cycle [5].

The *Plasmodium *parasite life-cycle is very complex, beginning with a larva-like structure (or sporozoite) being injected by the *Anopheles *mosquito during its bite in the search for a blood meal. The sporozoites then migrate to the liver and invade hepatocytes, where they rapidly reproduce and transform into thousands of pear-like structures (merozoites). During the asexual erythrocytic phase, which is responsible for the clinical manifestations of the disease, merozoites invade red blood cells (RBCs) very quickly through a process mediated by multiple receptor-ligand interactions [6]. A large number of parasite proteins associated with this type of interaction are stored in a set of specialized apical organelles known as rhoptries, micronemes and dense granules [7,8]. After initial contact with the RBC, the parasite redirects its apical pole over the erythrocyte membrane and sequentially releases the contents from micronemes, rhoptries and then the dense granules [9]. These molecular events lead to tight junction (TJ) and parasitophorous vacuole (PV) formation, as well as the biochemical and functional remodelling of host cell architecture [10].

A TJ is characteristic of members belonging to the phylum *Apicomplexa *and can be seen as a ring-shaped electro-dense structure by electron microscope; this connects to the parasite's actin-myosin motor [11] to propel the parasite within the nascent PV, where it will reside during the intraerythrocytic development cycle [12]. Several microneme- and rhoptry-derived proteins, such as reticulocyte-binding protein homologues (RH) [13,14], erythrocyte-binding ligands (EBL) and the MCP-1 protein [15] either form part or are associated with the TJ.

Apical merozoite antigen 1 (AMA-1) is derived from micronemes and is essential in invasion of most *Apicomplexa *studied so far [16,17]. It has been recently described that it is associated with proteins derived from the rhoptry neck in *Toxoplasma gondii*, such as RON-2, -4, -5 and -8 in the TJ. A TJ organizational model described by Besteiro *et al *in 2009 [18], proposed that the parasite directly inserts some RON proteins (also identified as AMA-1 associated proteins (AAPs)) into the host cell membrane, thus acting as additional *Tg*AMA-1 receptors. A clear interaction between the *Tg*RON2 C-terminal region and the AMA-1 ectodomain (forming a crucial bridge between *Tg*AMA-1 and the rest of the AAPs) has been recently demonstrated through different protein-protein interaction assays. Moreover, inhibition assays using recombinant proteins have shown that the RON2 and AMA-1 interaction is critical for the entry to host cells [19,20].

Previous comparative analysis between *T. gondii *and *P. falciparum *genomes has revealed the presence of homologues for *Tg*RON2, *Tg*RON4 and *Tg*RON5 proteins in *P. falciparum*: *Pf*RON2 (*Pf*14_0495), *Pf*RON4 (*Pf*11_0168) and *Pf*RON5 (MAL8P1.73), respectively. *Pf*RON2 [21], *Pf*RON4 [22] and *Pf*RON5 [23] are located in the rhoptry neck and co-immunoprecipitate with *Pf*AMA-1 [21,22,24,25]. Furthermore, the *Pf*RON2 protein and the *Pf*AMA-1 ectodomain interaction has already been characterized, as well as its importance for erythrocyte invasion, suggesting that the mechanism described in *T. gondii *could be conserved among different members of the phylum *Apicomplexa *[19].

Studies with parasite lines expressing *Pf*AMA-1 protein mutants have shown that the **Y251 **residue, located inside the hydrophobic channel, is absolutely essential for *Pf*AMA1/AAP complex formation [25]. Interestingly, an invasion inhibition antibody known as 4G2, that recognizes the domain II loop of *Pf*AMA-1 [26], prevents *Pf*AMA1/AAP complex assembly through steric hindrance and/or by inducing a *Pf*AMA-1 conformational change which interferes with the AAP binding site [25,27]. Likewise, the R1 peptide derived from a random phage display peptide library and known for being a powerful inhibitor of merozoite invasion of human RBCs [28] acts by binding to the *Pf*AMA-1 hydrophobic channel and blocking *Pf*AMA1-AAPs complex formation [29]. These data suggest that the interaction of a vaccine candidate molecule such as *Pf*AMA-1 with new rhoptry neck components is critical during invasion of erythrocytes and a better understanding of the molecular mechanisms involved in this process might thus help in developing new anti-malarial strategies.

Taking into account the importance and implication of RONs in different parasites belonging to the phylum *Apicomplexa *and based on previous studies carried out in *P. falciparum*, the identification and characterization of the first *P. vivax *rhoptry neck protein (*Pv*RON2), which is homologous to *Pf*RON2, are described in the present study. This protein is 2,204 amino acids-long (~220 kDa molecular mass), displaying an apical expression in *P. vivax *late schizonts, which suggests its role during invasion of target cells.

## Methods

### Bioinformatics methods

The search for a *Pf*RON2 homologous gene in *P. vivax *was carried out using the tBlastn tool in the *P. vivax *Sal-1 strain genome [30]. The sequence having the greatest score was selected as *pvron2 *putative gene. PlasmoDB and Sanger Institute [31] databases were scanned for *pvron2 *and *pfron2 *homologous genes in partial genomes from other *Plasmodium *species (*Plasmodium knowlesi*, *Plasmodium chabaudi*, *Plasmodium yoelii *and *Plasmodium berghei*). Identity and similarity values between *P. falciparum - P. vivax *and the other species were obtained with ALignX and ClustalW tools [32]. The presence of a signal peptide was assessed by using SignalP [33] and anchor regions were predicted using the PredGPI and TMHMM servers [34]. Repeat sequences and domains were predicted with the sequence tandem repeats extraction and architecture modelling software (XSTREAM, variable 'X'), the simple modular architecture research tool (SMART) and GlobPlot tools [35-37]. Bepipred tool [38] and ANTHEPROT software [39] were used for linear B epitope selection.

### Nucleic acids source and extraction

The *P. vivax *Colombia Guaviare 1 (VCG-1) strain was used as DNA, RNA and protein source. The strain was cultured through successive passes in *Aotus spp *monkeys from FIDIC's Primate Station in Leticia, Amazonas, as previously described [40] and according to the conditions established by the Ministry of the Environment's official Institute, Corpoamazonía (resolution 00066, September 13^th ^2006). Three to four mL of *P. vivax *VCG-1-infected monkey's blood were extracted; a schizont-rich sample was then obtained by discontinuous Percoll gradient (GE Healthcare, Uppsala, Sweden) according to a previously described protocol [41]. A Wizard genomic DNA purification kit (Promega, Wisconsin, USA) was used for genomic DNA extraction (gDNA) following the manufacturer's specifications. Total RNA was extracted by the Trizol method [42] and then treated with RQ1 RNase-free DNase (Promega, Wisconsin, USA). Five microlitres of RNA were used as cDNA synthesis template using the Superscript III enzyme (Invitrogen, Carlsbad CA) and oligo (dT) primers in a 5-min cycle at 65°C, followed by 60 minutes at 50°C and a final 15-min cycle at 70°C.

### Primer design, cloning and *pvron2 *gene sequencing

The *pvron-2 *nucleotide sequence (*PVX_117880*), reported in the PlasmoDB database, was used as template for designing three sets of primers with GeneRunner v3.05 software. PvRON2-pEXP-F1 5'-ATG ATA AGTA CAA GGG AGG CAA AA-3' and PvRON2-pEXP-R1 5'-ATA TCT TTT GTT TCT CGT CCT G-3' primers were used for amplifying **region I**, consisting of amino acids 18 to 742. PvRON2-pEXP-F2 5'-ATG AAC CCAT TAG TAT ATC ACG TG-3' and PvRON2-pEXP-R2 5'-CAG CAG TTT CAT CTTG GCC-3' were used for amplifying **region II**, consisting of amino acids 701 to 1560. **Region III **(amino acids 1517 to 2203) was amplified with PvRON2-pEXP-F3 5'-ATG ACC AGG GCT GAG AAA TTC G-3' and PvRON2-pEXP-R3 5'-CAC CTG TAT GCG GGC GTA-3'. Two primers were used for amplifying the PvAMA-1 ectodomain (43-487 amino acids): PvAMA-1D 5'-ATG CCT ACC GTT GAG AGA AGC A-3' and PvAMA-1R 5'-TAG TAG CAT CTG CTT GTT CG-3'.

PCR amplification was carried out using GoTaq Flexi DNA polymerase enzyme (Promega) in a 25 μL final reaction, according to manufacturer's instructions. Amplification conditions were as follows: a 7-min cycle at 95°C, followed by 35 cycles of 1 min at 58°C, 3 min at 72°C and 1 min at 95°C, and finally, a 10-min extension step at 72°C. Products were visualized on a 1% agarose gel and then purified with a Wizard PCR preps kit (Promega). PCR products obtained from cDNA were cloned in the pEXP5-CT/TOPO expression vector using TOPO TA cloning (Invitrogen, Carlsbad CA). Positive clones were analysed by enzymatic restriction and sequenced in an ABI PRISM 310 Genetic Analyser (PE Applied Biosystems).

### Peptide synthesis and polyclonal antibody production

Two linear B-cell epitope peptides were selected for producing polyclonal antibodies against the *Pv*RON2 protein based on the following parameters: (1) high average values for Parker's antigenicity, hydrophilicity and solvent accessibility obtained with Antheprot software [39], (2) high values in results obtained with the Bepipred tool (at default 0.35 threshold and 75% specificity) [38] and (3) selected peptides had to be located in different portions of the protein, with the aim of detecting different fragments in case the *Pv*RON2 protein was proteolytically processed. Selected peptides were synthesized by solid-phase peptide synthesis (SPPS) using the tert-butoxycarbonyl (t-Boc) strategy [43] and numbered according to our institute's serial numbering system as: 35519 (CG^734^YGRTRNKRYMHRNPGEKYKG^753^GC) and 35520 (CG^1674^KLQQEQNELNEEKERQRQEN^1693^GC).

Peptide 37870, derived from the N-terminal region of *Pv*AMA-1 protein (CG^23^RNQKPSRLTRSANNVLLE^40^GC), and 32416, derived from *Pv*RhopH3 protein (CG^792^SAGVGTVSTHSPATAARMGL^811^GC), were synthesized by SPPS. Peptide 37870 has been shown to be immunogenic in mice [44] and peptide 32416 has previously been used for polyclonal antibody production in rabbits, followed by localization experiments for the *Pv*RhopH3 protein [45]. Synthesized peptides were analysed by reverse phase high performance liquid chromatography (RP-HPLC) and MALDI-TOF mass spectrometry (Auoflex, Bruker Daltonics, Bremen, Germany). Cysteine and glycine were added to the N- and C-termini during synthesis to allow peptide polymerization. These peptides were inoculated in mice and the obtained sera were used for co-localization experiments as explained below.

Two New Zealand rabbits were selected (numbered 89 and 90) for obtaining polyclonal antibodies against *Pv*RON2 protein; they were negative for *P. vivax*-derived protein recognition by Western Blot. Each rabbit was subcutaneously inoculated with 500 μg of putative *Pv*RON2-derived peptide 35519 (rabbit 90) or peptide 35520 (rabbit 89), emulsified in Freund's complete adjuvant (FCA) on day 0. Booster immunizations on days 20 and 40 were administered using the same peptides emulsified in Freund's incomplete adjuvant (FCI). Rabbits' sera were collected on day 60 and used for further assays.

7-8 week old BALB/c strain mice were intraperitoneally (i.p.) immunized with 100 μg of peptide 37870 or peptide 32416, emulsified in FCA. Three boosters were given on days 30, 45 and 60 with 100 μg of FCI-emulsified peptide. These animals were bled 15 days after the last immunization and their sera were collected for further assays. Immunizations and animal bleeding were carried out following Colombian Ministry of Health recommendations for handling live animals used in research or experimentation.

### Immunoblotting and immunofluorescence

Saponin-treated parasite lysate was separated by 10% SDS-PAGE and proteins were then transferred to a nitrocellulose membrane. The membrane was blocked with a 5% milk solution in 0.05% PBS-Tween for one hour to eliminate unspecific binding. The membrane was cut into stripes for individual incubation with pre-immune and hyper-immune sera (anti-*Pv*RON2 polyclonal antibodies) in 1:20 dilution for 90 min, followed by incubation with phosphatase-coupled anti-rabbit IgG (PIERCE, Rockford, IL, USA) in a 1:5,000 dilution for 60 min. A BCIP/NBT kit (Promega) was used as a revealing solution, according to the manufacturer's instructions.

*Plasmodium vivax *VCG-1 thick smears were used for immunofluorescence assays and fixed with 4% v/v formaldehyde for 10 min. The slides were then permeabilized for 10 min with 1% v/v Triton and blocked with a 1% BSA/PBS solution at 37°C. The slides were washed several times with PBS and incubated with 300 μL of anti-*Pv*RON2 polyclonal serum (primary antibody) in a 1:40 dilution with either anti-*Pv*AMA-1 in a 1:20 dilution or anti-*Pv*RhopH3 in the same dilution for 60 min. Fluorescein-labelled anti-rabbit IgG (FITC) (Vector Laboratories, Burlingame, CA, USA) and rhodamine-labelled anti-mouse IgG (Millipore, Billerica, MA, USA) were used as secondary antibody for 60 min, followed by three PBS washes. Parasite nuclei were stained with a 2 μg/mL solution of 4',6-diamidino-2-phenylindole (DAPI) for 20 minutes at room temperature and fluorescence was visualized in a fluorescence microscope (Olympus BX51) using an Olympus DP2 camera and Volocity software (Perkin Elmer, Waltham, MA, USA).

## Results and Discussion

### *pvron2 *identification and orthologous genes

**Figure 1:**
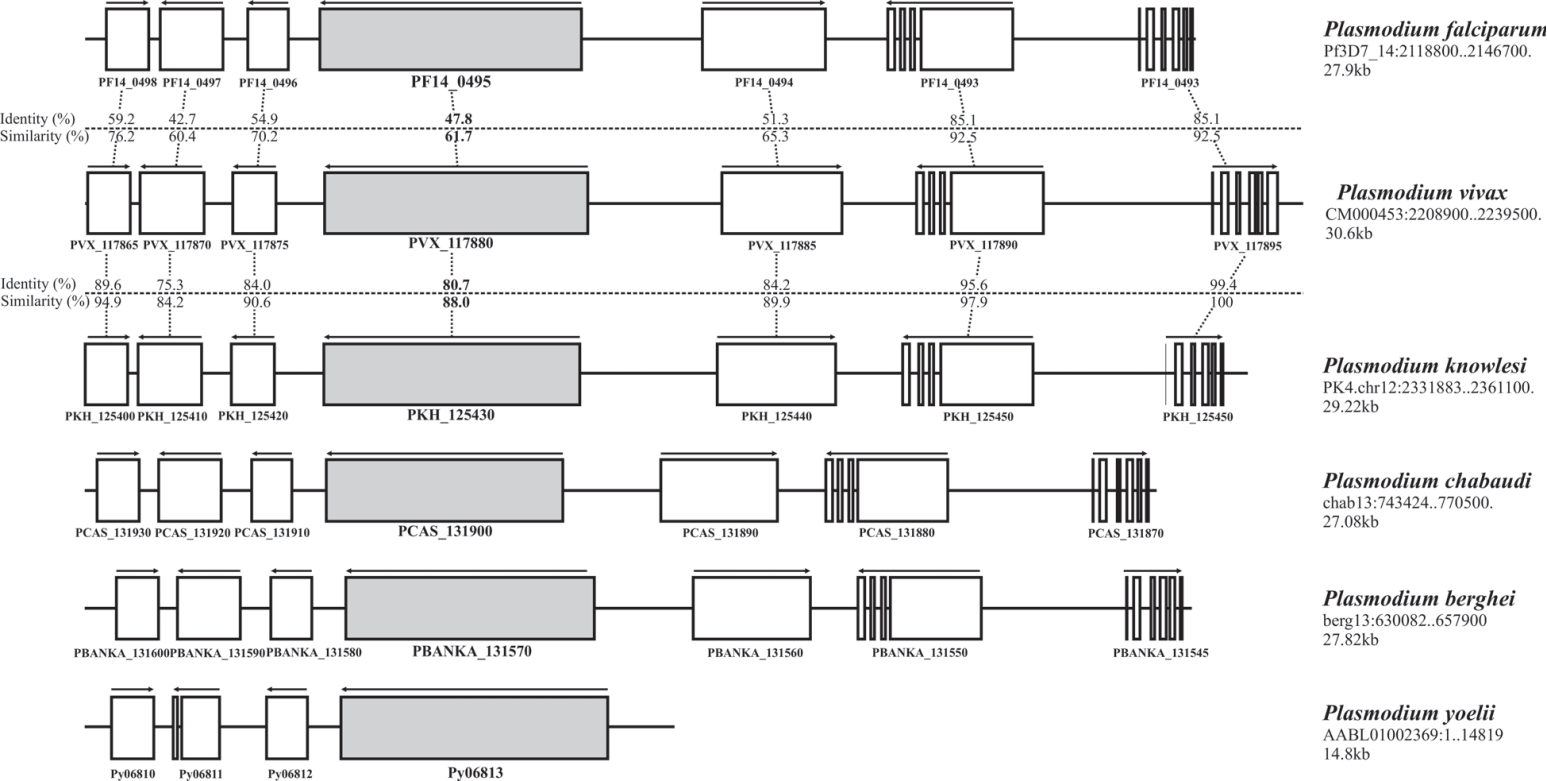
**Schematic representation of *ron2 *chromosomal localization (grey) and adjacent ORFs (white) in *Plasmodium falciparum, vivax, knowlesi, chabaudi, berghei *and *yoelii***. The accession numbers for each ORF in PlasmoDB are shown. The length of the represented chromosomal fragment and its localization within the genome in each species is shown on the right. Identity and similarity values at the amino acid level between *P. falciparum *and *P. vivax*, as well as between *P. vivax *and *P. knowlesi *are pointed out.

The *Pf*RON2 protein amino acid sequence (PF14_0495) was used as template for scanning the *P. vivax *complete genome, available in PlasmoDB (version 6.5), in the search for the homologous *Pv*RON2 encoding gene. tBlastn analysis revealed a nucleotide sequence having a high probability of containing the *pvron2 *gene located in reading frame -2 between 2,221,529-2,214,921 bp, contig CM000453. High similarity (61.7%) and identity (47.8%) values were found between *Pf*RON2 and *Pv*RON2 protein amino acid sequences, suggesting that these two proteins share a common origin. *pvron2 *neighbouring genes located upstream and downstream were also analysed, as well as their intron-exon organization; identity and similarity values were determined by comparing *P. falciparum *and *P. vivax *protein sequences (Figure 1). Similarity and identity values were found ranging from 60.4%-98.3% and 42.7-96.6%, respectively, in the analysed chromosomal region.

*Pf*RON2 and *Pv*RON2 orthologues were found in *P. knowlesi *(*Pk*RON2: PKH_125430), *P. chabaudi *(*Pc*RON2: PCAS_131900), *P. berghei *(PBANKA_131570) and *P. yoelii *(*Py*06813) when the *Pf*RON2 amino acid sequence was used as template for Blastp analysis for some *Plasmodium *species partial genomes. *Plasmodium *species *ron2 *genes were located in homologous chromosomal regions, as shown by their high similarity and identity values (35%-88% and 16%-75%, respectively) at amino acid level, similar ORF orientation and intron-exon pattern. *P. yoelii pyron2 *downstream genes (Figure 1) were excluded from analysis, given that this genome has not been completely assembled.

### *Pv*RON2 is encoded by a single exon and transcribed in blood-stage parasites

**Figure 2:**
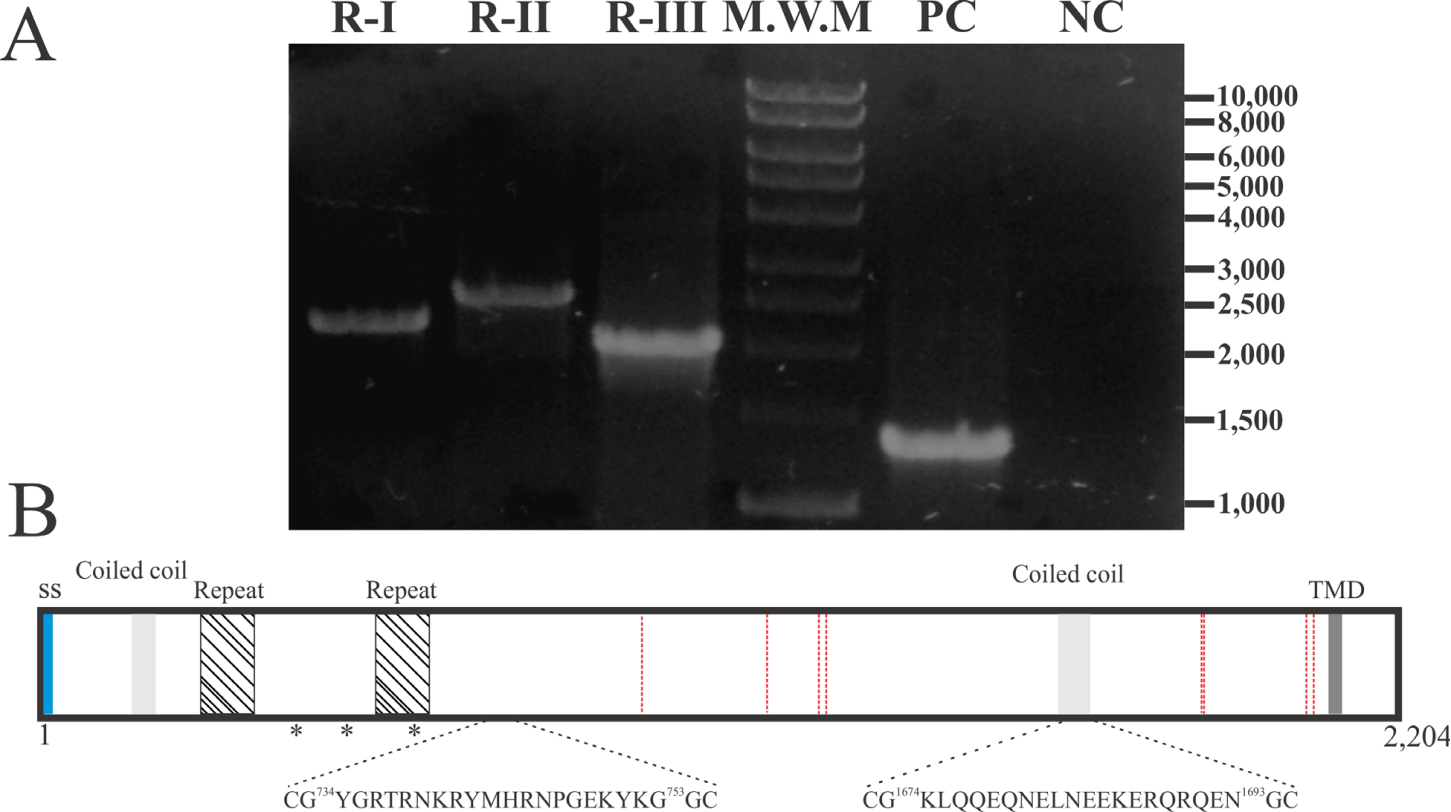
**cDNA amplification and *Pv*RON2 schematic representation**. **A**. PCR amplification from *pvron2 *gene RT-PCR product, with three sets of primers as described in the Materials and Methods section. Lane 1. *pvron2 *region I (~2,176 bp). Lane 2. *pvron2 *region II (~2,580 bp). Lane 3. *pvron2 *region III (~2,061 bp). Lane 4. molecular weight pattern. Lane 5. *Pv*AMA-1 ectodomain amplification (positive control). Lane 6. Negative control. **B**. *Pv*RON2 protein representation. The signal peptide is shown in blue, the transmembrane domain (TMD) in dark grey, coiled-coil motifs in light grey and red lines indicate conserved cysteines between *Pf*, *Pv*, *Pk*, *Pc*, *Pb *and *Py*. * represents polymorphic sites between Sal-1 (reference) and VCG-1 strains. The localization and sequence of inoculated peptides is marked.

*Plasmodium falciparum *transcriptome analysis revealed that *Pf*RON2 begins its transcription after 35 hours, reaching its maximum peak of expression 45 hours into the erythrocytic cycle [46]. PCR amplification of *Pv*RON2 encoding sequence confirmed the transcript's presence in *P. vivax *VCG-1 strain parasites during the blood stage (Figure 2A). This agreed with the results obtained from *P. vivax *transcriptome analysis which showed that *Pv*RON2 is transcribed between hour 35 (TP7) and 40 (TP8) in the intraerythrocytic cycle, similar to other proteins involved in invasion such as *Pv*MSP-1 [5]. When *pvron2 *gene gDNA and cDNA sequences were compared, obtained from the three amplification products overlapping by around 100 bp, both are identical, thus confirming that this gene consisted of a single 6,615bp exon. Recombinant clone sequences were analysed using CLC DNA Workbench (CLC bio) and the consensus sequence was deposited in the GenBank with the ID: HQ825321.

Two substitutions and the insertion of a nucleotide triplet were found when VCG-1 strain and Sal-1 reference strain nucleotide sequences were compared. Substitutions in positions 1,241 and 1,814 produced a change from valine to glycine (**V**414**G**) and histidine to proline (**H**605**P**), respectively. The addition of a glutamic acid (E)-encoding triplet (AAG) was found in position 1,487-1,489nt (residue 496). Interestingly, these changes were located in an interspecies variable region, spanning around residues 50 to 850 [21], suggesting that this region might be subjected to selective immune pressure.

### Bioinformatics analysis of *Pv*RON2 protein sequence

The *Pv*RON2 complete protein sequence in the VCG-1 strain consists of 2,204 residues having a putative hydrophobic signal sequence within its first 17 amino acids and a transmembrane domain (TMD) towards the C-terminus between residues 2,087-2,109. The RON2 protein has similar lengths in other species, ranging from 1,990 amino acids in *P. chabaudi *to 2,232 in *P. yoelii*, as well as a similar domain organization, including a signal peptide, a TMD and containing eight conserved cysteines (Figure 2B) probably related to common protein structural features.

*Pv*RON2 contains two coiled coil α-helical motifs (residues 145-184 and 1,651-1,703) (Figure 2B), characterized by seven amino acid repeats (**abcdefg**) _n _with hydrophobic residues located in positions **a **and **d**, and residues (generally polar) in the remaining sites which have been involved in protein-protein interactions. These coiled coil motifs have been identified in several important *P. falciparum *vaccine candidates such as LSA-1, MSP-3, MSP6 and MSP11 [47,48]; such motifs are recognized by naturally-acquired antibodies and are also immunogenic in mice [49]. Interestingly, peptide 35520 (containing part of the second coiled coil α-helical motif) has induced an antibody response in rabbits. Additionally, *Pv*RON2 has two tandem repeat (TR) regions located within the interspecies variable sequence. Eight 11 amino acid long repeats (GADGKGYGPYG) are located between residues 258 and 345, and the second tandem (GGYGNGGHE) is located between residues 542-628, having 9 repeats (Figure 2B). TRs were mostly found in RON2 sequences from different *Plasmodium *species and, even though the DNA and protein sequences from the repeats varied widely amongst RON2 proteins, there was close to 40% similarity between *Pv*RON2 and *Pk*RON2 repeats. Such similarity between *Pv *and *Pk *was in agreement with a close evolutionary relationship between simian malarial parasites and the human *P. vivax *parasite. TRs have been identified in different malarial antigens such as the *P. falciparum *circumsporozoite protein (CSP), the ring-infected erythrocyte surface antigen (*Pf*RESA) and the knob-associated histidine rich protein (KAHRP). These TRs could downregulate antibody isotype maturation and high-affinity antibody production in the specific case of malaria by acting as B-cell superantigens, predominantly inducing a polyclonal thymus-independent humoral response. T-independent antibody responses are usually short lived, predominantly composed of IgM and IgG3 and have low affinity, suggesting that these repeats are used during invasion to distract the immune response, acting as decoys or "smokescreens", thereby masking the critical epitopes [50,51]. Given all the above-mentioned data, it would be important to assess the functional and immunological implications of these repeat regions in *Pv*RON2.

### *Pv*RON2 is expressed in *P. vivax *schizonts

**Figure 3:**
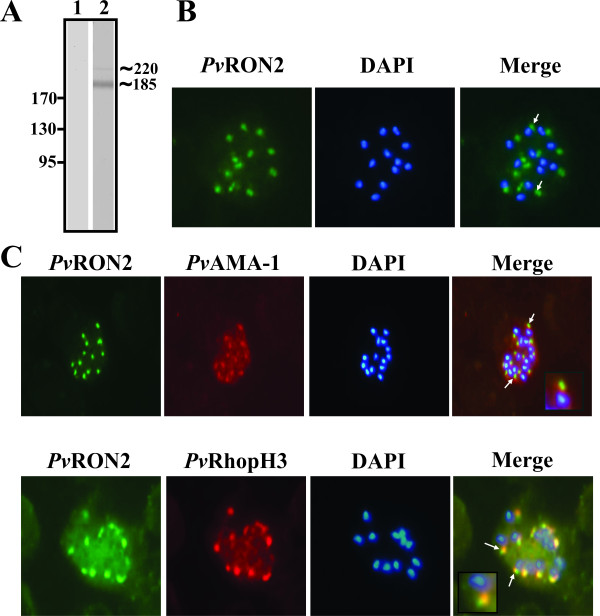
***Pv*RON2 expression and apical localization**. **A**. Anti-*Pv*RON2 rabbit polyclonal antibodies detected two bands at around ~220 and ~185 kDa in parasite lysate by Western blot. Lane 1: pre-immune serum. Lane 2: hyper-immune serum. **B**. *P. vivax *schizonts incubated with anti-*Pv*RON2 polyclonal antibodies and revealed with FITC-labelled anti-rabbit IgG (green). Parasite nuclei were stained with DAPI (blue). **C**. Co-localization study: schizonts were simultaneously incubated with anti-*Pv*RON2 and anti-*Pv*AMA-1 (top) or anti-*Pv*RhopH3 (bottom) and detected with FITC-labelled anti-rabbit and with rhodamine-labelled anti-mouse. Arrows indicate the typical dotted pattern displayed by apical organelles.

Polyclonal antibodies were produced against the protein by immunizing rabbits with polymeric *Pv*RON2-derived peptides to assess *Pv*RON2 expression and cellular localization in *P. vivax *schizonts. Polyclonal antibodies detected two bands at around ~220 kDa and ~185 kDa (Figure 3A), suggesting that *Pv*RON2 can undergo proteolytic processing, by contrast with that reported for *Pf*RON2 [21]. The predicted size for *Pv*RON2 (240 kDa) is slightly larger than that obtained from mobility on SDS-PAGE (220 kDa). Interestingly, similar behaviour has been described for *Tg*RON2, suggesting anomalous migration [52].

In many cases, it has been found that rhoptry proteins are initially synthesized as pre-proteins and maturate during transport [53]. It could be hypothesized that such cleavage could serve to activate *Pv*RON2 by revealing a functional domain or releasing the protein of parasite surface or RBC membrane to allow successful invasion. Additionally, pulse-chase analysis has shown that *Tg*RON2 is expressed as a pro-protein (~150 kDa) which is cleaved to produce a ~120 kDa mature protein. Even though it is not known which specific proteases act in *Tg*RON2 maturation, it has been suggested that this protein can be cleaved by subtilisin 2 (*Tg*SUB2) [18]. Studies carried out with important *P. falciparum *adhesins located on merozoite membrane, micronemes or rhoptries, such as AMA-1, merozoite surface protein (MSP), EBL, RBL and thrombospondin-related anonymous protein (TRAP) families, contain a putative rhomboid cleavage site within their TMD and putative SUB-2 cleavage sites. COS-7 cell system studies have revealed that A1427 residue substitution in the EBA-175 protein has prevented *Pf*ROM4-mediated shedding, avoiding the release of EBA-175 from the merozoite surface [54]. Similarly, substituting the GA motif (residues which destabilize α-helices) closest to the TMD extracellular end abolished specific cleavage (also predicted as the site required for rhomboid recognition). Interestingly, *Pv*RON2 sequence analysis revealed a putative rhomboid cleavage site between 2,101-2,104; this agreed with the fact that sera recognized two fragments from the protein, but additional studies are needed for assessing the importance of such processing, as well as the identity of the responsible protease.

Immunofluorescence analysis of *P. vivax *schizonts showed that *Pv*RON2 had a dotted pattern typical of apical organelles, such as rhoptries and micronemes (Figure 3B). To examine their localization in detail, dual labelling was performed using mouse polyclonal antibodies against *Pv*AMA-1 and *Pv*RhopH3. It was found that there was no co-localization between the *Pv*AMA-1 protein (microneme marker) and *Pv*RON2 (Figure 3C), suggesting that *Pv*RON2 is not present in micronemes. By contrast, there was a small area of central localization between *Pv*RhopH3 (rhoptry bulb marker) and *Pv*RON2 suggesting that even though *Pv*RON2 is located in the rhoptries, it is not located in the rhoptry bulb, probably forming part of the rhoptry neck, as has been described for *Pf*RON2 and *Tg*RON2 proteins [18,21]. Recently, a study that characterized the timing of expression and subcellular location of *Plasmodium *homologues in some *T. gondii *rhoptry proteins showed that *P. berghei *RON2 protein is located in merozoite and sporozoite rhoptries, and presents a timing of expression comparable to the one found in RAP2/3. These data strongly suggest an essential role of RON2 protein during the invasion and infection establishment in sporozoites [55].

## Conclusions

As has been shown in the present study, RON2 is a highly conserved protein among different *Plasmodium *species. *pvron2 *gene consists of a single exon and is transcribed and expressed in schizonts rhoptries at the end of the erythrocytic cycle. Its similarity to *Pf*RON2 (which forms a complex with PfAMA-1), as well as its localization and expression time during the schizont stage suggest a similar role in host cell invasion for *Pv*RON2, as that attributed to *Pf*RON2.

## Competing interests

The authors declare that they have no competing interests.

## Authors' contributions

GAP carried out bioinformatics analyses, molecular biology assays and wrote the initial manuscript. HC synthesized and purified the peptides used for rabbit and mice immunizations and analysed data. LCP carried out immunoassays. MAP evaluated and coordinated assays, and revised the final manuscript. All authors read and approved the final manuscript.
